# Neonatologist performed echocardiography (NPE) in Italian neonatal intensive care units: a national survey

**DOI:** 10.1186/s13052-019-0721-z

**Published:** 2019-10-22

**Authors:** Iuri Corsini, Benjamim Ficial, Stefano Fiocchi, Federico Schena, Irma Capolupo, Rosa Maria Cerbo, Manuela Condò, Daniela Doni, Simona La Placa, Salvatore Porzio, Katia Rossi, Sabrina Salvadori, Marilena Savoia, On Behalf of Study Group of Neonatal Cardiology of the Italian Society of Neonatology

**Affiliations:** 1 Neonatal Intensive Care Unit, Division of Neonatology, Careggi University Hospital of Florence, Florence, Italy; 2 Neonatal Unit, Azienda Ospedaliera Universitaria Integrata di Verona, Verona, Italy; 3 Neonatologia e Terapia Intensiva Neonatale, Ospedale Valduce, Como, Italy; 4 Neonatal Intensive Care Unit, Fondazione IRCCS Cà Granda Ospedale Maggiore Policlinico di Milano, Milan, Italy; 5 Neonatal Intensive Care Unit, Ospedale Pediatrico Bambino Gesù, Rome, Italy; 6 Neonatal Intensive Care Unit, Fondazione IRCCS Policlinico San Matteo, Pavia, Italy; 7 Neonatal Intensive Care Unit, Ospedale A. Manzoni, Lecco, Italy; 8 Neonatal Intensive Care Unit, FMBBM San Gerardo, Monza, Italy; 9 Neonatal Intensive Care Unit, AOUP Giaccone, Palermo, Italy; 10 Neonatal Section, San Michele Hospital, Maddaloni, NA Italy; 11 Neonatal Intensive Care Unit, Policlinico di Modena, Modena, Italy; 12 Neonatal Intensive Care Unit, Azienda Ospedaliera-Università di Padova, Padova, Italy; 13 Neonatal Intensive Care Unit, Azienda Ospedaliera Universitaria S Maria della Misericordia, Udine, Italy

**Keywords:** Neonatologist performed echocardiography, Neonatal cardiology, Functional echocardiography, Neonatal intensive care

## Abstract

**Background:**

Neonatologist performed echocardiography (NPE) has increasingly been used to assess the hemodynamic status in neonates. Aim of this survey was to investigate the utilization of NPE in Italian neonatal intensive care units (NICUs).

**Methods:**

We conducted an on-line survey from June to September 2017. A questionnaire was developed by the Italian neonatal cardiology study group and was sent to each Italian NICU.

**Results:**

The response rate was 77%. In 94% of Italian NICUs functional echocardiography was used by neonatologists, cardiologists or both (57, 15 and 28% respectively). All the respondents used NPE in neonates with patent ductus arteriosus and persistent pulmonary hypertension, 93% in neonates with hypotension or shock, 85% in neonates with perinatal asphyxia, 78% in suspicion of cardiac tamponade, and 73% for line positioning.

In 30% of center, there was no NPE protocol. Structural echocardiography in stable and critically ill neonates was performed exclusively by neonatologists in 46 and 36% of center respectively.

**Conclusions:**

NPE is widely used in Italian NICUs by neonatologists. Structural echocardiography is frequently performed by neonatologists. Institutional protocols for NPE are lacking. There is an urgent need of a formal training process and accreditation to standardize the use of NPE.

## Background

There is an urgent need for adequate monitoring hemodynamic conditions in preterm and term infants requiring intensive care, in order to identify neonates at risk of hypoperfusion and long-term neurological sequelae, in addition to individualize treatment. Several methods have been proposed but echocardiography has assumed a primary role in this field.

Previously echocardiography was performed by pediatric cardiologists to diagnose and monitor congenital heart diseases (CHD). More recently, echocardiography has increasingly been used by neonatologists as an adjunct in the clinical assessment of the hemodynamic status in neonates [1]. The term “functional echocardiography” (f-echo) has been introduced to describe the use of echocardiography performed by neonatologist for cardiovascular assessment [2, 3].⁠ There is a growing evidence that f-echo is a useful clinical tool in the identification of hemodynamic instability and in guiding treatment [4, 5]⁠. In 2011 the American society of echocardiography (ASE), the European association of echocardiography (EAE), and the association for European paediatric cardiology (AEPC) published practice guidelines and recommendations for training in targeted neonatal echocardiography (TNE), also known as f-echo [6]⁠. More recently, a working group of the European society for paediatric research (ESPR) and the European society for neonatology (ESN) wrote a consensus statement on f-echo, namely Neonatologist Performed Echocardiography (NPE) [7]⁠, taking into account the previous TNE recommendations. Furthermore a series of review articles discussing the current status of NPE has lately been produced by the ESPR “special interest group on NPE” on key topics of f-echo [8]⁠. Data on utilization of echocardiography in the neonatal intensive care unit (NICU) were previously reported [9–11]⁠, but, to the best of our knowledge, no data are available from Italy. Aim of our survey study was to investigate the utilization of NPE in Italian NICUs.

## Methods

We conducted a prospective cross-sectional survey study from June to September 2017.

### Questionnaire

Four members (I.C., B.F., S.F., F.S.) of the study group of neonatal cardiology (SGNC) of the Italian society of neonatology (SIN) developed a 37-item questionnaire according to the CHERRIES method for internet e-surveys [12]. The questionnaire was divided into 9 sections: epidemiological and organizational characteristics of the center; NICU link with pediatric cardiology service; echocardiographic equipment; imaging storage and reporting; infection control, cardiorespiratory and thermal stability; practice of structural echocardiography; practice of f-echo; parameters of f-echo used cot-side (data on f-echo and patent ductus arteriosus (PDA) were investigated in a different survey that will be published shortly); training needs. All questions were loaded to the Google Forms Website, a free tool for creating online survey forms (https://docs.google.com/forms/u/0/), and were proofread. Prior to distribution, the survey was pilot-tested to identify potential inaccuracies by the other 9 members of the SGNC and modified accordingly, as reported in literature [13, 14]⁠. The questionnaire required approximately 30 min to be completed.

### Web-based survey

A cover letter containing a hyperlink to the survey was sent to the directors of Italian NICUs and to one attending neonatologist at each site with expertise in NPE, based on the database of the SIN and the SGNC. To ensure only one response for center a unique survey link was assigned to each institution. To prevent incomplete answers, the survey form could be submitted only when completed.

The first email was sent in June 2017 and a reminder in September 2017. Those who did not replied were subsequently contacted by e-mail and/or phone call. No financial rewards were offered for participating in the survey. Completion of the questionnaire implied consent to participate in the survey. The identity of each participant was kept confidential throughout the data collection and analysis. The missing responses of some centers defined the study as ‘voluntary inquiry with presence of non-respondents’.

### Data analysis

Google Forms automatically converted every questionnaire into Excel files (Microsoft, Seattle, WA). I.C. and B.F checked every questionnaire for potential inconsistencies throughout this process of conversion. We compared data of centers that assist less than 50 very low birth weight (VLBW) per year vs center that assist more than 50 VLBW per year to test significative differences. The threshold was chosen arbitrarily.

Continuous variables were tested for normality using the Shapiro-Wilk test and presented as means and standard deviation (SD) or median and interquartile range (IQR) as appropriate. Categorical variables were presented as proportions. Comparisons between subgroups were conducted using a Student t test or a Mann-Whitney U test as appropriate. Categorical variables were compared using the χ^2^ or Fisher.

## Results

### Epidemiological and organizational characteristics of the centers

The overall survey response rate was 77% (88/114). Centers had a median of 8 intensive care cots (IQR 6–10).

### Link with a pediatric cardiology service

Sixty-three percent of respondents (55/88) had a pediatric cardiology service on site. Fourteen percent of respondents (12/88) had a pediatric cardiac surgery service.

Significant differences in pediatric cardiology and cardiac surgery availability were found comparing centers with low and high patients’ volume (i.e. < 50 or ≥ 50 VLBW per year): a pediatric cardiologist service was available in 51 and 74% of centers respectively (*p* < 0.05) and a pediatric surgeon was available in 2 and 26% of centers respectively (*p* < 0.05).

Seventy percent of centers (62/88) had a neonatologist or a cardiologist on call on a 24-h basis, trained to perform echocardiography in neonates. A significant difference was found between centers with low and high volume: 55 and 86% respectively (p < 0.05).

In 86% of centers (76/88) neonatologists performing echocardiography had a pediatric cardiologist they could contact on a 24/7 basis, in case they need a specialist opinion or in case of doubts. However, pediatric cardiologists could not constantly provide an echocardiography on site in a timely manner.

### Echocardiographic equipment

Ninety-four percent of centers (83/88) had a dedicated cardiac ultrasound machine available in the unit. The echocardiographic equipment had high frequency probes (> 7 MHz) in 98% of cases, B-mode (2D) and M-mode in 100%, pulse wave doppler in 98%, continuous wave doppler in the 95% and tissue doppler imaging in 56%. Only 49% of centers used electrocardiogram tracing during echocardiography.

### Imaging storage and reporting

Eighty-four percent of centers did not have a digital image archiving system and in 60% of cases the report of the exam was not standardized.

### Infection control

Measures taken in order to limit the spread of infections were hand washing (100%), disinfection of the probe (92%) and use of sterile gel (26%).

### Cardiorespiratory and thermal stability

To reduce the impact on the body temperature and cardiorespiratory stability the measures adopted were: heated gel (26%), use of probe for continuous detection of body temperature of the baby (58%), interruption of the examination in case of desaturation and / or bradycardia (97%).

### Practice of structural echocardiography

**Fig. 1:**
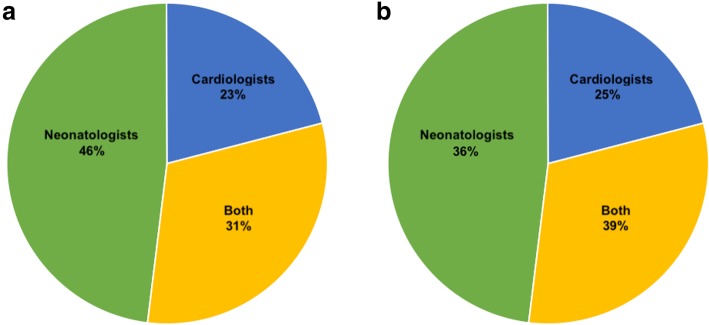
Practitioner performing echocardiographic assessment in order to exclude CHD in stable (**a**) and in critically ill patient (**b**)

In hemodynamically stable neonates with suspicion of CHD, (eg baby with a systolic murmur), the person performing the first echocardiography was a neonatologist, a pediatric cardiologist or both in the 46, 23 and 31% of centers respectively (Fig. 1a). In hemodynamically unstable neonates with suspicion of CHD (eg. baby with cyanosis or shock) the person performing the first echocardiography was a neonatologist, a pediatric cardiologist or both in the 36, 25, and 39% of centers respectively (Fig. 1b).

In 63% of respondent centers the first cardiac ultrasound was always a comprehensive echocardiography aimed at excluding a CHD. There is no significant difference between high and low volume centers.

The median percentage of neonatologists with the skills to perform a structural echocardiography in the respondent centers was 18% (IQR 8–25%). A significant difference was found between low and high volume centers: 14% (8–21%) and 21% (8–30%) respectively (*p* < 0.05).

### Practice of functional echocardiography

**Fig. 2:**
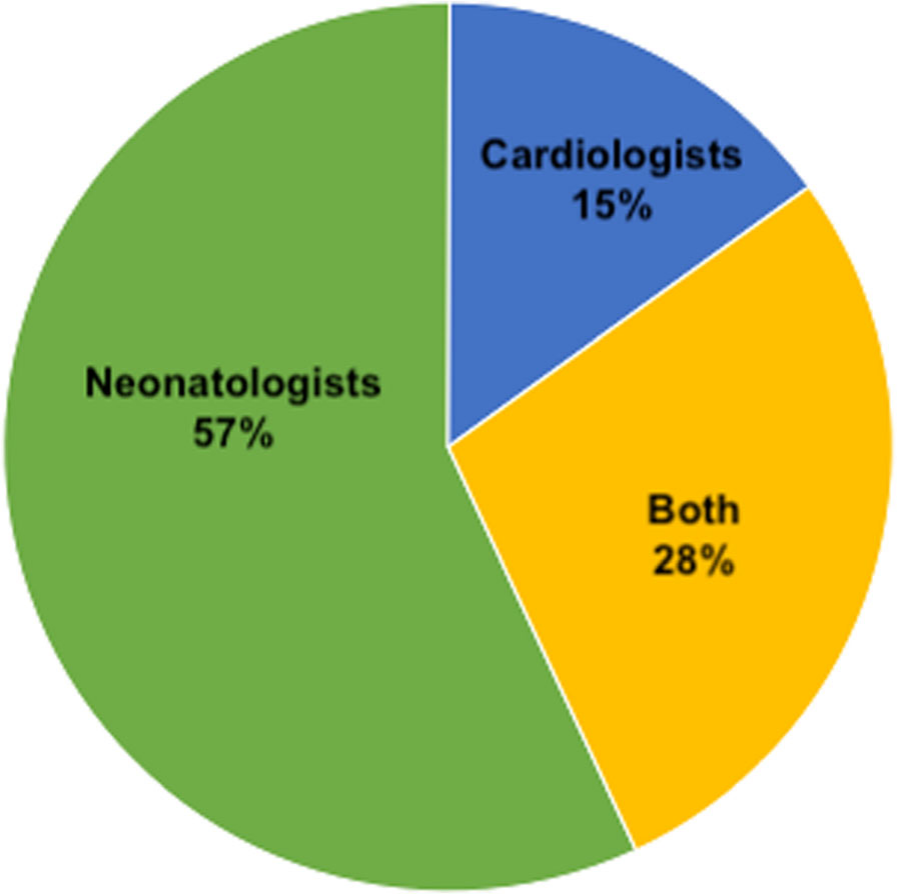
Practitioner performing functional echocardiography

Ninety-four percent (83/88) of respondent centers used f-echo, which was performed by neonatologist, cardiologist or both in 57, 15 or 28% of centers respectively: no significant difference was found between low and high volume centers (Fig. 2).

**Fig. 3:**
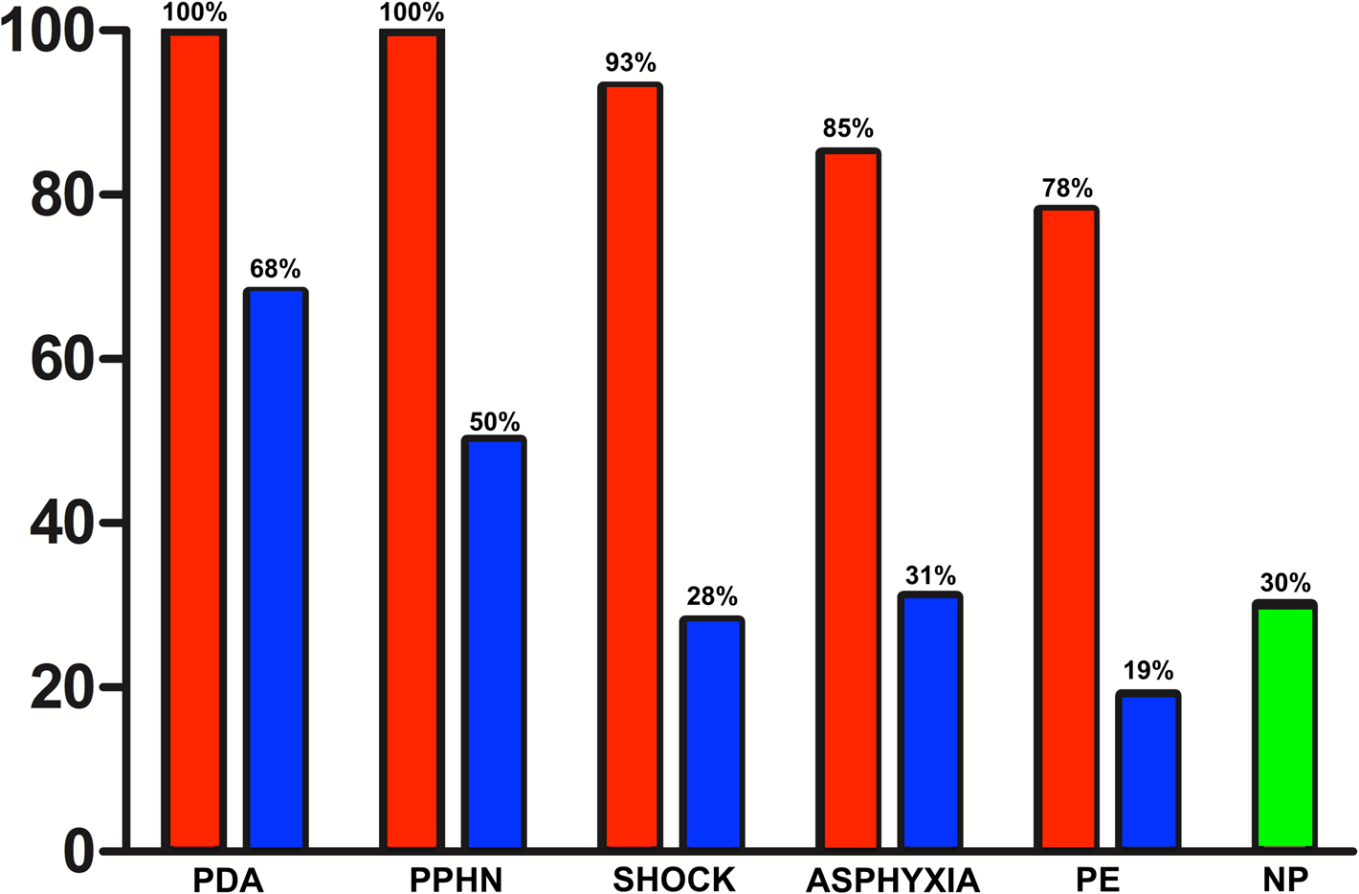
Proportion of centers that use NPE divided for neonatal condition. Red columns: Proportion of centers that use NPE divided for neonatal condition. Blue Columns: Proportion of centers using standardized protocol for NPE divided for neonatal condition. Green Column: Proportion of centers that use NPE without any protocol. PDA: Patent ductus arteriosus; PPHN: Persistent Pulmonary Hypertension of the Newborn; PE: Pericardial Effusion; NP: No protocol

The most commonly reported indications included PDA evaluation (100%), persistent pulmonary hypertension of the newborn (PPHN) (100%), hypotension and/or shock (93%), perinatal asphyxia (85%), suspected cardiac tamponade (78%) and placement of central vascular catheters (73%) (Fig. 3).

Protocols to standardize its use were reported for PDA in 68% of the centers, for PPHN in 50%, for hypotension and / or shock in 28%, for perinatal asphyxia in 31%, for suspected cardiac tamponade in 19%. In 30% of centers there was no institutional protocol regulating the utilization of f-echo (Fig. 3).

Detailed data on the modality of the echocardiographic evaluation of the duct are discussed in a specific survey of the Italian SGNC *(data currently submitted).*

### Parameters of NPE used at the cot-side

We investigated the echocardiographic parameters used to evaluate cardiac function in different clinical scenarios according to the currently available guidelines [6, 7].

Eye-ball evaluation of left ventricular systolic function was performed in 52% of centers whereas fraction shortening (FS) and ejection fraction (EF) were used in 73 and 72% of centers respectively.

Neonatologists used more frequently “eye-ball” evaluation of contractility compared to cardiologists (55% vs 46% respectively).

Measures of left ventricular systolic function were less commonly adopted: left ventricular output (LVO) was measured in 56% of centers and superior vena cava flow (SVCf) in 48%. Neonatologists used more frequently LVO and SVCf compared to cardiologists (59 and 46% vs 44 and 40% respectively).

Left ventricle diastolic function was evaluated in 85% of centers: measures used were left atrium dimension in 69% of centers, mitral E- and A-wave velocity and E/A ratio in 62%, pulmonary veins flow in 28%.

Moving to the right ventricular function, 55, 52, and 11% of centers evaluated tricuspidal annular systolic excursion (TAPSE), right ventricular output (RVO), and fractional area change (FAC) respectively. TAPSE was used with the same frequency by neonatologist and cardiologists (approximately 54%), whereas FAC was more used by cardiologists (19% vs 9%).

Right ventricle diastolic function was evaluated in 51% of centers measuring tricuspidal E- and A-wave velocity and E/A ratio.

Pulmonary artery pressure was measured in 95% of centers with the classic method of detecting the tricuspid regurgitation jet flow velocity (m/sec)^2^ × 4. Other measurements used were the end-systolic inter-ventricular septum shape in parasternal short axis view (69%), early peak velocity of the pulmonary regurgitation (51%) or acceleration time / ejection time ratio (26%). No significant difference was found in the utilization of the above mentioned parameters between cardiologists and neonatologists.

In case of pericardial effusion 91% of centers quantified the effusion measuring the diastolic dimension of the effusion. Twenty percent of centers used respiratory variation of the atrio-ventricular transvalvular flow.

### Training in echocardiography

Ninety percent of neonatologists who answered to the questionnaire did their training in echocardiography attending in pediatric cardiology centers and in neonatal units renowned for their expertise in functional echocardiography.

Fifthy-one percent of respondents attended a post-graduate master in pediatric cardiology, whereas 11% specialized in cardiology. However, in almost all cases, training in f-echo consisted of theoretical and practical courses. The composition of the latter was not standardized and highly variable.

The development of an accredited training pathway in NPE was considered essential by 61% of respondents.

## Discussion

We investigated the utilization of NPE in Italian NICUs, being echocardiography increasingly used by neonatologists as an adjunct in the clinical assessment of the hemodynamic status in neonates.

Previously reported data showed international variation in the utilization of f-echo, ranging from an almost total coverage (e.g. France, Australia and New Zealand) to a minority of NICUs having f-echo capability (e.g. USA, Canada) [9, 11, 15–17]⁠. To the best of our knowledge, no data were available from Italian NICUs.

The survey received a high response rate (77%), almost entirely from level III neonatal units and the remaining from level IV. Individual phone call positively raised response rate after e-mail reminder.

We found that the vast majority of Italian neonatal units practiced NPE (94%). Like in other European countries, there is no legal impediment to doctors to use ultrasound imaging, this probably was, among others, an essential factor that allowed widespread utilization of functional echocardiography in Italy [3, 15]⁠.

F-echo was mainly performed by neonatologists rather than cardiologists and the clinical indications were: PDA, PPHN, perinatal asphyxia, hypotension/shock, cardiac tamponade, assessment of line position. These data were similar to the ones from other European countries, Australia, and New Zealand [3, 15]⁠.

Almost all units had an exclusive ultrasound machine and the echocardiographic equipment fulfilled the requirements of the ESPR/ESN Consensus Statement (i.e. high frequency probe, B-mode, M-mode, pulse wave doppler, continuous wave doppler). Instead, ECG tracing during echocardiography, digital image archiving system and standardized reporting, that were considered by the ESPR/ESN recommendations essential components in every scan, were still not widespread. More effort should be done to implement these components of NPE in order to meet the European standards and to improve practice.

Both ESPR/ESN and UK recommendations proposed measures to prevent infections and to maintain cardiorespiratory and thermal stability while scanning. Our data showed adequate attention to these issues. Nevertheless, a simple measure such as the use of heated gel was not widely adopted and it should be implemented [7, 18]⁠. Although some studies showed that echocardiography could be performed in critically ill neonates without significant cardiorespiratory or thermal instability, we did not investigate specifically this topic [8, 19]. Further prospective studies should be carried out to ensure that NPE is safely performed among NICUs.

In the past, lack of universally accepted guidelines of NPE practice together with the little evidence on how to integrate these data in the clinical decision-making led to differences in practice between centers [11]⁠. The latter was confirmed by our data, showing that practice of NPE was markedly heterogeneous between Italian NICUs and even within the same center. In fact, in 30% of centers there was no institutional protocol to regulate the practice of echocardiography. Where present, protocols for PDA and PPHN management were more common, compared to other clinical scenarios.

Echocardiographic parameters used to evaluate the cardiac function in different clinical scenarios were performed according to the currently available guidelines [6, 7]⁠. They included: evaluation of LV systolic and diastolic function, RV function, pulmonary pressure, assessment of atrial-level shunt, PDA, systemic blood flow and pericardial effusion.

Normal values where not clearly defined by guidelines and not investigated in our survey. Lack of universally renowned normal values for echo parameters contributes to increase the heterogeneous application of f-echo in daily clinical practice.

Minimal differences were found between measurements used by neonatologists and cardiologists. Neonatologists more frequently assessed cardiac output than cardiologists (SVCf and LVO) and chose eyeball evaluation of left ventricular systolic function, whereas cardiologists preferred quantitative measurements (SF and EF). A continuous collaboration between cardiologists and neonatologists would promote more consistency in functional assessments and better understanding of hemodynamic compromise. However, considering the number and the specificity of functional assessments required in a busy neonatal unit, in addition to the need to integrate echo and clinical data in clinical decision-making, functional echocardiography would probably remain the domain of neonatologists with adequate training [1–3, 20].

The utilization of NPE poses a unique challenge: structural normality cannot be assumed because around 0.5–1% of all newborn infants have CHDs [16]⁠. TNE and ESPR/ESN guidelines recommended that the first evaluation should always be a comprehensive study, in order to safely identify babies with structural abnormalities [6, 7]⁠. Our data showed that only in 63% of cases the first evaluation was a comprehensive study aimed at excluding a CHDs, in addition to functional assessment; from our survey it cannot be ascertained if normal heart structure were confirmed afterwards.

There is an urgent need to train Italian neonatologists to practice functional echocardiography following the ESPR/ESN recommendations, in order to ensure safety of practice. This can not be overemphasized, in particular because, according to our data, a considerable number of neonatologists performed echocardiography in critically ill neonates even with suspicion of CHDs, in order to identify congenital abnormalities [6, 7, 16]⁠. ESPR/ESN guidelines recognize that many neonatologists across Europe undertake additional clinical roles in diagnosis and follow-up of CHD; it could be argued that lack of pediatric cardiology centers within the same institution would favor this, an event that occurs especially in centers with lowest volume of work. According to our survey, the majority of neonatal units had a pediatric cardiology service on site (63%) and the vast majority of centers had a pediatric cardiologist they could contact on a 24/7 basis (86%). Nevertheless, pediatric cardiologists could not constantly provide an echocardiography on site in a timely manner.

We feel that a close collaboration with pediatric cardiologists should be further implemented among Italian NICUs, according to the ESPR/ESN guidelines, in order to ensure safety and accuracy in identifying babies with critical CHDs [7]. Further studies should be carried out to investigate the percentage of misdiagnosed CHDs, if any, when the first ultrasound evaluation is performed by neonatologists. Due to the limits of our survey study (i.e. respondent biases), we could not address this topic.

However, available data showed that, in the presence of a close collaboration between neonatologist and pediatric cardiologists during training and beyond, there was concordance of echocardiographic findings between neonatologists and cardiologists, even in the presence of structural abnormalities [21].

The majority of neonatologists did their training in echocardiography attending in pediatric cardiology centers and in neonatal units renowned for their expertise in functional echocardiography. More than half of respondents had high level training, however median percentage of neonatologists with the skills to perform a structural echocardiography in the respondent centers was up to 55% at most. Courses offered both nationally and internationally had non-homogeneous programs, greatly lacking in practical training. The development of an accredited training pathway in NPE was perceived essential by the majority of respondents.

Our data highlight many issues related to acquirement of competency, health care organization and training, similarly to those observed in other countries [8, 16, 17]⁠. In response to these needs the SGNC, in agreement with the SIN, is currently designing and implementing a formalized and accredited training program in NPE. A close collaboration is retained with the Italian society of pediatric cardiology (SICPED), that supported and endorsed this project. Aims are to offer adequate training to meet the standards required by the ESPR/ESN recommendations, to ensure safety and to uniform practice of NPE.

This was a survey study and data were self-reported, therefore they may be open to respondent biases.

## Conclusions

Neonatologist performed echocardiography had a widespread use in Italian NICUs, even so the majority of neonatal units had no institutional protocol to regulate the practice of echocardiography.

In addition to hemodynamic assessment, Italian neonatologists are currently playing a significant role in the first evaluation of neonates with suspicion of CHDs.

As in the vast majority of the other countries, there is an urgent need to ensure standardization of clinical practice guidelines and to design and implement a formalized and accredited training program, in the effort to pursue quality assurance and patient safety.

## Supplementary information

**Additional file 1.** List of Neonatal Units respondent to the survey.

## Data Availability

The data that support the findings of this study are available from Italian Society of Neonatology but restrictions apply to the availability of these data, which were used under license for the current study, and so are not publicly available. Data are however available from the authors upon reasonable request and with permission of Italian Society of Neonatology.

## Abbreviations

AEPC: Association for European paediatric cardiology
ASE: European association of echocardiography
CHD: Congenital heart disease
EAE: European association of echocardiography
EF: Ejection fraction
ESN: European society for neonatology
ESPR: European society for paediatric research
FAC: Fractional area change
f-echo: Functional echocardiography
FS: Fraction shortening
IQR: Interquartile range
LVO: Left ventricular output
NICU: Neonatal intensive care unit
NPE: Neonatologist performed echocardiography
PDA: Patent ductus arteriosus
PPHN: Persitent pulmonary hypertension of the newborn
RVO: Right ventricular output
SD: Standard deviation
SGNC: Study group of neonatal cardiology of the Italian society of neonatology
SIN: Italian society of neonatology
SVCf: Superior vena cava flow
TAPSE: Tricuspidal annular systolic excursion
TNE: Targeted neonatal echocardiography
VLBW: Very low birth weight
